# Dance with the Devil: Stress Granules and Signaling in Antiviral Responses

**DOI:** 10.3390/v12090984

**Published:** 2020-09-04

**Authors:** Nina Eiermann, Katharina Haneke, Zhaozhi Sun, Georg Stoecklin, Alessia Ruggieri

**Affiliations:** 1 Division of Biochemistry, Mannheim Institute for Innate Immunoscience (MI3), Medical Faculty Mannheim, Heidelberg University, 68167 Mannheim, Germany; Nina.Eiermann@medma.uni-heidelberg.de (N.E.); Katharina.Haneke@medma.uni-heidelberg.de (K.H.); Georg.Stoecklin@medma.uni-heidelberg.de (G.S.); 2 Department of Infectious Diseases, Molecular Virology, Center for Integrative Infectious Disease Research (CIID), University of Heidelberg, 69120 Heidelberg, Germany; Zhaozhi.Sun@med.uni-heidelberg.de

**Keywords:** virus, stress granules, stress response, innate immune response, PKR, G3BP1, antiviral signaling

## Abstract

Cells have evolved highly specialized sentinels that detect viral infection and elicit an antiviral response. Among these, the stress-sensing protein kinase R, which is activated by double-stranded RNA, mediates suppression of the host translation machinery as a strategy to limit viral replication. Non-translating mRNAs rapidly condensate by phase separation into cytosolic stress granules, together with numerous RNA-binding proteins and components of signal transduction pathways. Growing evidence suggests that the integrated stress response, and stress granules in particular, contribute to antiviral defense. This review summarizes the current understanding of how stress and innate immune signaling act in concert to mount an effective response against virus infection, with a particular focus on the potential role of stress granules in the coordination of antiviral signaling cascades.

## 1. Introduction

Viruses depend on the host translation apparatus to express viral proteins. By hijacking and redirecting ribosomes, translation factors, and RNA binding proteins (RBPs), viruses modulate the cellular translatome in favor of their needs and virus progeny production. In rare cases, viruses such as hantaviruses, myoviruses, haloarchaeal viruses, and giant viruses encode viral proteins that substitute for translation initiation, elongation, and termination factors, transfer RNAs (tRNAs), aminoacyl-tRNA synthetases [1,2,3,4,5,6], and—as lately identified via metagenome analysis—also ribosomal proteins [7]. Although the use of the host-encoded translation machinery saves coding capacity, the ensuing dependency on host factors to accomplish this critical step of the viral life cycle makes viruses vulnerable. To counteract virus reproduction, cells have evolved highly specialized stress sensors that detect viral products and actively suppress both host and viral translation. Hence, limiting the access, availability, or activity of the translation apparatus can be considered an effective defense mechanism and integral component of the antiviral response.

Immune sensors detect viral nucleic acids as non-self through specific features, termed pathogen-associated molecular patterns (PAMPs), among these incoming viral DNA and RNA genomes and viral replication intermediates. The activation of immune sensors elicits an antiviral state through the production of interferons (IFNs) and pro-inflammatory cytokines [8,9,10]. In addition, the accumulation of viral double-stranded (ds) RNA in the cytosol and of viral proteins in the endoplasmic reticulum (ER) triggers stress sensors such as protein kinase R (PKR) and PKR-like endoplasmic reticulum kinase (PERK), respectively [11]. Once activated, these kinases initiate the integrated stress response (ISR) by phosphorylating the alpha subunit of the eukaryotic translation initiation factor-2 (eIF2α), which delivers the Methionine initiator tRNA (tRNAi^Met^) to the small 40S ribosomal subunit. As a consequence, translation initiation is stalled and polysomes disassemble [12].

The assembly of stress granules (SGs) is an integral part of host stress responses, frequently observed upon infection with DNA or RNA viruses. SGs form by a cytosolic phase separation event, through which stalled mRNAs are separated from the remaining cytosol together with translation factors. SGs were primarily suggested to serve as storage and triage areas for mRNAs that can easily be reengaged with polysomes in order to resume translation once the stress is resolved [13,14]. However, the abundance of RBPs and signal transducing factors in SGs has broadened our perspective on the role of SGs in recent years. SGs are now considered to be signaling platforms that contribute to the coordination of cellular processes during stress, including apoptosis, cell growth, metabolic control, and antiviral defense [15,16,17,18]. It is therefore not surprising that viruses not only escape translational repression through various mechanisms, but also interfere with the assembly of SGs and repurpose SG proteins for their own replication [19,20,21,22]. The diversity of these viral strategies highlights the importance of the stress response, and SGs in particular, in antiviral defense.

Whether SGs as a whole or single proteins within SGs contribute to the antiviral response is still an open question whose answer might depend on the virus type, the cell type, and the resulting host–virus interactions. Recent advances in the purification of SG components have opened the route to investigate which proteins and mRNAs are selectively recruited to SGs during viral infections. Here, we aim to summarize the current understanding of the translational stress response in the context of virus infection and cover the various connections between the stress response and innate immune response, which are intertwined events that act in concert to establish an antiviral state. Emphasis will be given to the potential role of SGs in the initiation and coordination of antiviral signaling events.

## 2. General Mechanisms of Translation Control under Homeostasis and Virus Infection

Regulation of gene expression at the level of translation is a central control mechanism that enables rapid and reversible changes in protein levels, both spatially and temporally. In addition to essential roles in several biological processes, including cell growth, differentiation, and apoptosis [23,24,25], translational control plays a major role in the host stress response to virus infection [26,27].

### 2.1. Translation Initiation, a Key Step in the Regulation of Protein Synthesis

Mammalian protein synthesis is primarily regulated at the level of translation initiation, which requires recruitment of the small ribosomal subunit to the mRNA, scanning along the mRNA 5′ untranslated region (UTR), recognition of the start codon, and subsequent joining of the large ribosomal subunit [28]. These steps are regulated by upstream open reading frames (uORFs), RNA modifications, RNA structures and different RBPs, which together dictate mRNA translation initiation efficiency [29,30].

Most cellular mRNAs contain a cap structure at their 5′ end, composed of an inverted 7-methylguanosine connected to the mRNA via a 5′-5′-triphosphate bridge. Most 5′ ends are additionally methylated at the ribose 2′*O* position of the +1 ribonucleotide (cap1), and in many cases, also of the +2 ribonucleotide (cap2). This feature is not only important for mRNA translation and stability, but also for the distinction between self from non-self mRNAs [31]. Indeed, several single-stranded (ss) RNA viruses that replicate in the cytosol, including flaviviruses, picornaviruses, coronaviruses, and poxviruses, encode their own 2′*O*-methytransferases to escape recognition by host immune sensors [32]. Initially, the 5′ cap is bound by the eIF4F complex, which consists of the cap-binding protein eIF4E, the RNA helicase eIF4A, and the scaffold protein eIF4G. Subsequently, the 40S small ribosomal subunit together with the initiation factors eIF1, eIF1A, eIF5, eIF3, and the eIF2–GTP–tRNAi^Met^ ternary complex is loaded onto the mRNA 5′ end, forming a scanning-competent 48S preinitiation complex (PIC). The PIC then scans the 5′ UTR until it recognizes a start codon within a favorable sequence context, leading to hydrolysis of the eIF2-bound GTP. This crucial step results in the release of eIF2-GDP, leaving the initiator tRNAi^Met^ base-paired with the AUG start codon [33].

### 2.2. Repression of Translation Initiation upon Environmental Stress

Cells respond to unfavorable conditions such as amino acid starvation, ER stress, oxidative stress, hypoxia, UV irradiation, and virus infection by rapid attenuation of translation initiation rates, mainly through controlling (i) the eIF2–GTP–tRNAi^Met^ ternary complex and (ii) the cap-binding complex. Thereby, the translation of mRNAs not critical for cell survival, e.g., housekeeping mRNAs, is strongly repressed during stress in favor of mRNAs encoding survival factors, repair enzymes, and stress-regulatory proteins [23,24].

The first mechanism involves four eIF2α-kinases, which are activated by a wide range of extrinsic and intrinsic stressors, and phosphorylate the α subunit of eIF2 at Ser51 [12]. This prevents release of eIF2 from the GTP-GDP exchange factor eIF2B, thereby impairing regeneration of the ternary complex and slowing down protein synthesis [34]. The heme-regulated inhibitor (HRI) senses oxidative stress, osmotic stress, heat shock, and heme-depletion. PKR is activated by cellular and viral dsRNAs. PERK, a transducer of the unfolded protein response (UPR), is activated by the accumulation of misfolded proteins in the ER. Finally, the general control nonderepressible 2 (GCN2) is mainly activated by limited amino acid availability and UV stress. As a common feature, eIF2α-kinases were found to be activated by stress-induced oligo- or dimerization and autophosphorylation [11]. The pool of the GTP-bound ternary complex is regenerated by dephosphorylation of eIF2α through protein phosphatase 1 (PP1), whose catalytic subunit is recruited to eIF2α via a specific regulatory subunit. In unstressed cells, the constitutive repressor of eIF2α phosphorylation (CReP), a constitutively expressed regulatory subunit of PP1, maintains low levels of eIF2α phosphorylation [35], especially at the ER [36]. Under stress conditions, the PP1 regulatory subunit growth arrest and DNA damage inducible protein 34 (GADD34) is both transcriptionally and translationally upregulated, antagonizing eIF2α phosphorylation and translational arrest imposed by eIF2α-kinase activation [37,38,39,40].

The second mechanism is based on regulating the activity of the eIF4F cap-binding complex, which controls cap-dependent translation. Certain types of stress, such as nutrient deprivation and hypoxia, lead to inhibition of the mammalian target of rapamycin (mTOR) complex 1 (mTORC1), the key kinase that regulates the phosphorylation state of 4E binding proteins (4E-BPs) [24]. In their unphosphorylated form, 4E-BPs have an increased affinity for the cap-binding protein eIF4E and outcompete eIF4G, preventing efficient recruitment of eIF3 and the 40S small ribosomal subunit to the 5′ end of the mRNA [41]. Thereby, inhibition of mTORC1 leads to a reduction in cap-dependent translation.

A subset of cellular mRNAs contains linear motifs and secondary structures that enable protein synthesis under stress conditions through alternative modes of translation initiation, which are independent of eIF2α and/or eIF4F. These include (i) transcripts containing short uORFs [42], mostly encoding for survival-related and stress-effector proteins, e.g., the transcription factors activating transcription factor 4 (ATF4) and the CCAAT/enhancer-binding protein homology protein (CHOP), as well as GADD34. (ii) Approximately 10% of all cellular mRNAs are suggested to contain internal ribosome entry sites (IRES) and hence, do not require eIF4F binding, but instead, rely on IRES trans-acting factors (ITAFs) for efficient translation initiation [43,44,45]. IRES elements were originally discovered in viruses belonging to the Picornaviridae family [46,47]. Even though their activity and efficiency is still under debate, numerous cellular IRES elements have been reported and characterized, particularly in genes involved in apoptosis such as the X-linked inhibitor of apoptosis protein (XIAP) [48] and B-cell lymphoma-2 (Bcl-2) [49], and more recently, in genes involved in cell growth and proliferation such as mTOR [50] and c-Src [51]. The existence and involvement of a growing number of cap-independent initiation mechanisms in eukaryotes, including (iii) N6-methyladenosine (m^6^A)-dependent translation [52,53,54], and (iv) the use of alternative cap-binding complexes such as eIF4FH under hypoxic conditions or eIF4FM in response to stress or proliferative cues, respectively [55], are indisputable.

The impact of translational repression, especially on RNA viruses, is highlighted by the fact that many evolved alternative initiation strategies to translate their genome independently of eIF2α, including the use of IRES elements [56] and the possibility to switch from cap-dependent to cap-independent translation [57,58,59,60].

### 2.3. From Translational Suppression to SG Formation

When translation initiation is blocked under stress conditions, stalled translation preinitiation complexes accumulate. At the same time, translation elongation continues and polysomes disassemble as a consequence of ribosome run-off. Upon acute and strong inhibition of translation initiation, non-translating mRNAs condensate together with RBPs into microscopically visible, non-membranous cytosolic SGs through a liquid–liquid phase separation event [61]. Phase separation is likely initiated by the appearance of long stretches of mRNA not covered by ribosomes, which engage in RNA–RNA interactions [62] and serve as scaffolds for numerous RBPs that promote phase separation through their intrinsically disordered regions. RBPs with an essential role in nucleating SGs include RasGAP-associated endoribonuclease 1 (G3BP1), T cell internal antigen 1 (TIA1), TIA1-related protein (TIAR), Caprin1, and fragile X mental retardation protein (FMRP) [63]. Recently, ubiquitin-associated protein 2-like (UBAP2L) [64,65,66,67], cold shock domain containing E1 (CSDE1), and proline-rich coiled-coil 2C (PRRC2C) were added to the growing list of SG-nucleating proteins [64]. Overexpression of single proteins such as TIA1 or G3BP1 can drive SG formation, even in the absence of stress [68,69], indicating that a shift in the equilibrium between solubilizing and aggregation-prone proteins is sufficient to induce phase separation of the cytosol.

Interestingly, SGs were found to consist of a more stable inner core, stabilized by direct protein–protein and RNA–protein interactions, and a dynamic shell-like outer layer that is characterized by multiple, multivalent low-affinity interactions between proteins and RNAs [70]. SGs are very dynamic structures, which rapidly assembly under stress conditions and disassemble within minutes when cells recover from stress [71,72]. Depending on the type of stress, SGs vary in size and number, and differ with respect to some of their mRNA and protein constituents. During oxidative stress, for example, SGs move in a microtubule-dependent manner and grow in size by fusion [71,73].

Given their dynamic nature, it is not surprising that proteins and mRNAs shuttle in and out of SGs in the seconds to minute range [71,74,75], showing that SG components are in constant exchange with the cytosol. SGs also exchange proteins and mRNAs with processing bodies (PBs), a different type of cytosolic RNA granule that contains RNA degrading enzymes and functions in mRNA silencing [76]. SGs and PBs often exist in close proximity and may represent different stages of an “mRNP cycle” [13].

A peculiar type of SG dynamic was discovered by our laboratory upon chronic hepatitis C virus (HCV) infection, which leads to recurring cycles of SG assembly and disassembly, following an oscillation of translational on- and off-states. The stochastic nature of SG assembly and disassembly is controlled by the antagonistic action of PKR and GADD34, which repeatedly phosphorylate and dephosphorylate eIF2α. This oscillating stress response is widely observed upon infection with RNA viruses including Newcastle disease virus (NDV) and Sendai virus (SeV) [77]. Our current understanding is that oscillating SGs represent a long-term strategy by which infected cells suppress viral protein production and replication intermittently, while enabling host protein production in between SG phases. Moreover, rapid SG oscillations seem to correlate with enhanced cell survival, suggesting a role in balancing the burden of antiviral defense with cellular homeostasis.

## 3. Stress Kinases—Mediators of Viral Translational Inhibition

Translation inhibition is an important pillar of the antiviral response. Viruses evolved multiple strategies to target all steps of translation initiation in order to evade host-induced translational shutoff and promote the synthesis of their own proteins. Since these strategies have been covered in several excellent reviews [26,27,56], we will focus here on how stress kinases contribute to the induction of the ISR upon virus infection and how viruses directly target these kinases. While global translation suppression upon infection with RNA viruses is primarily mediated by PKR, activation of other stress kinases, alone or in combination with PKR, has been reported. They can be considered as additional antiviral barriers, especially when viruses have evolved strategies to counteract PKR.

### 3.1. Protein Kinase R (EIF2AK2)

Among the four eIF2α-kinases, PKR, formerly called DAI, contributes to the sensing of viral infection and is the object of extensive research [78,79,80]. PKR is an IFN-induced effector protein [81] that is activated upon binding of dsRNA molecules [82]. The binding of dsRNA to the two dsRNA-binding motifs (dsRBD) within the PKR N-terminal domain promotes a structural reorientation, which allows for PKR dimerization and subsequent activation by autophosphorylation of PKR C-terminal kinase domain. These structural rearrangements are required for binding and phosphorylation of eIF2α [82,83,84,85]. One of the earliest molecules found to activate PKR was a hairpin loop of the hepatitis delta virus genome, within the self-cleaving ribozyme region [86,87,88]. A similar hairpin structure was discovered in the human immunodeficiency virus (HIV) genome within the transactivation-response region [89]. In general terms, viral activators of PKR represent dsRNA regions longer than 30 bp [85,86], including the genome of dsRNA viruses like rotaviruses [90], dsRNA replication intermediates of positive and negative ssRNA viruses [91,92] as well as dsRNA products from antiparallel transcription of DNA viruses such as vaccinia virus (VACV) [93]. The activation of PKR by short-stem loops has been suggested to be 5′ triphosphate-dependent [94,95], though this observation is discussed controversially [96,97].

In recent years, PKR was shown to have functions, beyond the detection of viral dsRNA, in cellular processes such as mitosis and apoptosis [98,99], in metabolic as well as autoimmune diseases [100,101,102,103], and in long-term memory [104,105,106]. Cellular activators of PKR include mitochondrial dsRNAs [99], dsRNAs derived from inverted Alu repeats [98,107], non-coding small nucleolar RNAs [100], ribotoxin-induced or damaged rRNAs as well as unmodified rRNAs or tRNAs [108,109,110]. RNAs that inhibit PKR have also been identified, e.g., circular RNAs [103] and the human non-coding RNA 886 [111,112].

Viruses have evolved a multitude of strategies to counteract PKR. For instance, many RNA viruses encode proteins that bind and shield dsRNA from PKR detection, exemplified by VACV E3L [113], influenza A virus (IAV) accessory protein NS1 [114], Middle East respiratory syndrome coronavirus accessory protein 4a [115,116], reovirus sigma 3 protein [117], and Ebola virus protein VP35 [118,119]. Other viruses such as human parainfluenza virus type 3 (HPIV3) sequester viral RNA in inclusion bodies to avoid detection by PKR [120]. Herpes simplex virus (HSV) 1 and 2 encode an endoribonuclease, the virion host shutoff protein that degrades RNA to avoid PKR activation early during infection [121,122,123]. Negative ssRNA viruses such as measles virus (MV), influenza virus, and SeV avoid detection by PKR by making sure that only a small number of dsRNA replication intermediates accumulate in the cytosol [91]. This is achieved by the virally encoded accessory protein C, which attenuates the copy-back mechanism of the viral RNA polymerase during replication [124,125,126].

Other viruses suppress PKR by encoding proteins that directly inhibit the kinase function, e.g., HCV non-structural protein NS5A [127], Japanese encephalitis virus (JEV) non-structural protein NS2A [128], human cytomegalovirus protein TRS1 [129], and Kaposi’s sarcoma-associated herpesvirus lytic protein ORF57 [130]. An interesting variation of this theme is the expression of small regulatory RNAs by some DNA viruses, which antagonize PKR activation by competing with dsRNA binding. Examples include the adenovirus VAI RNAs and the Epstein–Barr virus transcripts EBER-1 and EBER-2 [131,132,133], all of which bind to but do not activate PKR.

An alternative strategy adopted by some viruses is mimicry. Baculovirus protein PK2, for example, is an inactive kinase with homology to PKR, which leads to the formation of inactive PKR heterodimers [134,135]. The VACV K3L protein is an eIF2α homologue, which competes with eIF2α for PKR binding and thereby reduces eIF2α phosphorylation [113]. Finally, PKR can be targeted to proteasomal degradation upon infection with Rift Valley fever virus by the NSs protein [136,137,138].

### 3.2. PKR-Like Endoplasmic Reticulum Kinase (EIF2AK3)

During their life cycle, many RNA viruses perturb or hijack ER functions by (i) remodeling ER membranes to form viral replication and assembly sites [139], (ii) utilizing and competing for the host protein glycosylation machinery [140,141], and (iii) encoding viral proteins with viroporin function that alter ER calcium homeostasis [142]. Virus-imposed interference with ER functions causes ER stress and induces the three signaling branches of the UPR through activation of IRE1-XBP1, ATF6, and PERK, whereby infected cells aim to reestablish ER homeostasis. PERK, as one player of the UPR, prevents protein production, hence alleviating the ER burden [143]. For certain viruses, PERK activation was reported to be beneficial, notably for NDV [144] and Seneca Valley virus [145]. Here, PERK-induced autophagy is essential for viral replication. However, in multiple cases, PERK has adverse effects for viral replication. Transmissible gastroenteritis virus (TGEV) but also West Nile virus (WNV) and Langat virus replication, for instance, are inhibited via PERK signaling [146,147,148].

Hence, it is not surprising that viruses counteract PERK activity. NDV, e.g., mediates PERK cleavage [149], whereas dengue virus (DENV) infection was found to inhibit PERK activation by a yet unknown mechanism [150]. The VACV K3L protein was reported to reduce PERK activity in vitro [151], and similar results were obtained for the HCV-encoded glycoprotein E2. This protein induces ER stress on the one hand [152], but acts as a pseudo-substrate and thereby inhibits PERK activation on the other hand [153]. These examples illustrate that viruses strongly interfere with ER functions but at the same time, often try to prevent the consequent activation of PERK. Interestingly, PERK can also get activated by viral proteins such as JEV-encoded protein NS4B, which induces PERK dimerization and is known to be important for the pathogenesis of encephalitis [154]. This is consistent with the fact that PERK’s implication in virus infection might differ depending on the virus type or infection stage.

### 3.3. General Control Nonderepressible 2 (EIF2AK4)

GCN2 is usually activated under conditions of amino acid starvation through its histidyl-tRNA synthetase-related domain that binds uncharged tRNAs [155]. This domain also binds to viral RNA genomes in vitro, notably those of sindbis virus (SINV), poliovirus, and HIV-1, and in vivo studies confirmed that infection with SINV and HIV-1 leads to activation of GCN2, eIF2α phosphorylation, and translational inhibition of viral RNA [156,157]. Furthermore, HIV-1 induces protease-mediated cleavage of GCN2, indicating that GCN2 has an antiviral function that the virus tries to suppress [157]. This notion is supported by the observation that GCN2-deficient or GCN2-mutant mice are more susceptible to infection with SINV [156], murine cytomegalovirus, and human adenovirus [158].

In the future, it will be important to further characterize the mechanism behind GCN2 activation in virus-infected cells since this might also be an indirect consequence of virus- or IFN-γ-induced amino acid deprivation.

### 3.4. Heme-Regulated Inhibitor (EIF2AK1)

A direct implication of HRI in virus infection was reported only recently for its fish homologues. In flounder cells (*Paralichthys olivaceus*), an upregulation of HRI was observed upon infection with *Scophthalmus maximus* rhabdovirus and poly(I:C) treatment, at both the mRNA and protein levels [159]. Furthermore, overexpression of HRI in orange-spotted grouper (*Epinephelus coioides*) resulted in an increase in eIF2α phosphorylation and inhibition of red-spotted-grouper nervous necrosis virus (RGNNV) replication [160]. Both studies suggested that HRI might have a similar function to PKR in fish.

Normally, HRI is activated by high intracellular levels of reactive oxygen species (ROS) and other imbalances of redox homeostasis, e.g., those induced by arsenite [161]. Several viruses interfere with mitochondrial, peroxisomal, and ER functions and can thereby lead to ROS production, as observed during infection with influenza virus [162], flaviviruses such as DENV, WNV, and JEV [163], as well as chronic viruses such as HCV and hepatitis B virus [164,165,166,167], HIV [168], and poxviruses [169]. In many cases, ROS production represents a necessary event for viral replication or other processes such as capping [170,171]. At the same time, viruses manipulate the antioxidative defense system to maintain ROS levels in a range that is optimal for their purpose, without inducing cell death [172]. However, in contrast to fish, there are no reports, to our knowledge, showing an activation and implication of HRI upon virus infections in mammalian cells.

## 4. Stress Kinases—Mediators of Innate Immune Signaling

Beside their function in translational control, stress kinases were reported to be involved in a multitude of immune modulatory processes. In the following section, we summarize the currently known connections between the immune and stress sensing pathways.

### 4.1. Innate Immune Signaling Pathways

Innate immune sensors represent the first line of defense against viral infection. They span a wide range of receptor families: RIG-I-like receptors (RLRs) including retinoic acid-inducible gene I (RIG-I) and melanoma differentiation-associated protein 5 (MDA5), Toll-like receptors (TLRs), C-type-lectin receptors, NOD-like receptors, AIM2-like receptors, and cytosolic DNA sensors (CDSs) such as ZBP-1, GMP-AMP synthase (cGAS), DDX41, and IFI16. Due to differences in their cellular localization (plasma membrane, endosomes, cytosol) and binding preference (proteins, dsDNA, ssRNA, dsRNA), the diversity of these sensors enables the detection of a large panel of PAMPs. Upon ligand binding, receptors initiate specific intracellular signaling cascades via different signaling adapters. RLRs signal via the mitochondrial-associated adaptor protein MAVS, CDSs via the ER adaptor molecule stimulator of interferon response CGAMP interactor (STING), and endosomal TLRs via TRIF and MyD88 [173,174,175,176,177].

Notably, all these signaling pathways converge on the activation of the transcription factors IFN-regulatory factor (IRF) 3 and 7 by the TANK-binding kinase 1 (TBK1) and nuclear factor-κ-B (NF-κB) by the IκB kinase (IKK) complex [9,10,178,179]. Nuclear translocation of IRF3/7 phosphorylated forms leads to the induction of type I IFN genes. Upon secretion, IFNs act in an autocrine and paracrine manner, engage with IFN receptors and induce Janus kinase/signal transducers and activators of transcription (JAK/STAT) signaling [180], leading to the transcriptional activation of hundreds of IFN-stimulated genes (ISGs) with antiviral activity [181], including PKR. Thereby, type I IFNs establish an antiviral state that is critical for containment of viral infections. In parallel, phosphorylation of IκB by the IKK complex results in its dissociation from NF-κB and its degradation. In turn, NF-κB translocates into the nucleus where it initiates transcription of numerous pro-inflammatory cytokines and chemokines (e.g., interleukin (IL)-1, IL-6, IL-8, tumor necrosis factor α (TNF-α)), as well as anti-apoptotic proteins (e.g., Bcl-2, cFLIP, Fas) that promote cell survival [182,183].

The expression of cytokines and IFNs is further controlled at the posttranscriptional level via mitogen-activated kinases Erk1/2 and p38 MAPK signaling, through the downstream kinases Mnk1/2 and MK2. The phosphorylation of eIF4E by Mnk1/2, for instance, upregulates translation of IκB and IRF-1 [184,185]. Moreover, MK2-dependent phosphorylation of Tristetraprolin (TTP), an RBP that binds to AU-rich elements (AREs) located in the 3′ UTR of many cytokine and IFN mRNAs, prevents rapid degradation of these mRNAs and further promotes their translation [186,187,188].

### 4.2. Impact of Stress Kinases on Innate Immune Signaling Pathways

**Figure 1:**
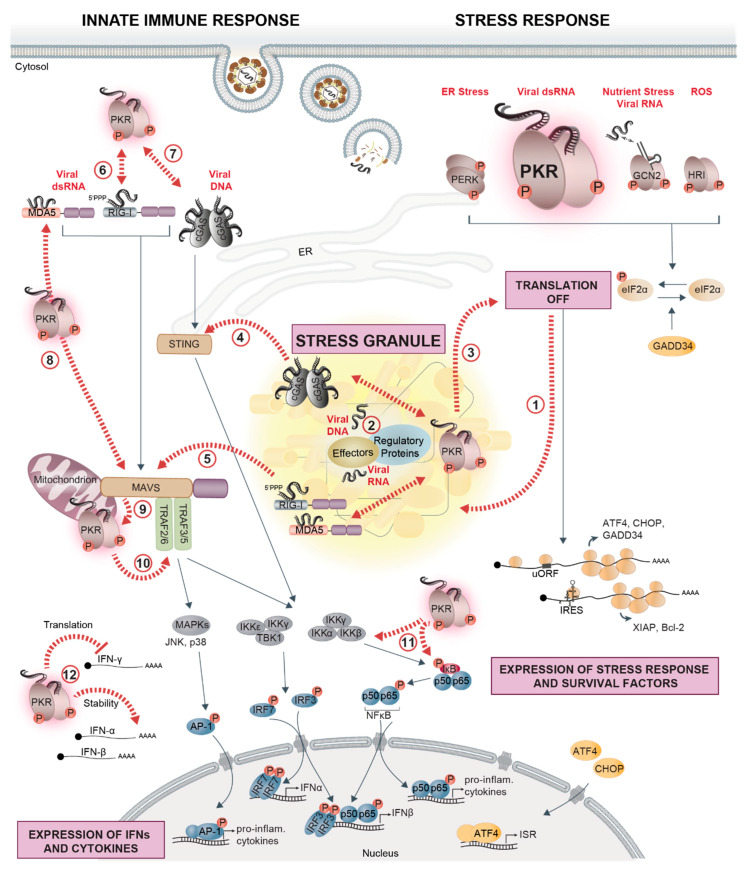
PKR at the crossroads of virus sensing, innate immune signaling, and stress response pathways. Cells evolved several cytosolic sentinels that detect virus infection and initiate defense mechanisms, including innate immune and stress responses. Cytosolic nucleic acid sensors RIG-I, MDA5, and cGAS signal via the mitochondrial adapter protein MAVS and the ER-adapter protein STING, triggering downstream signaling cascades via the IKK complex (IKKγ, IKKα, IKKβ) and IKKε/IKKγ/TBK1. The IKK complex phosphorylates the NF-κB (p50, p65) inhibitor IκB, whose degradation enables the nuclear translocation of NF-κB and transcriptional activation of IFN-β and pro-inflammatory cytokines. TBK1, on the other hand, phosphorylates IRF3/7, whose nuclear translocation mediates the transcriptional activation of IFN-α/β (left side). Cytosolic dsRNA that accumulates during viral replication is sensed by the stress kinase PKR. As a consequence, phosphorylation of eIF2α strongly represses the translation of most cellular mRNAs, while the translation of factors related to the stress response (ATF4, CHOP, and GADD34) or cell survival (XIAP and Bcl-2) is selectively favored (right side). The other eIF2α-kinases, GCN2, PERK, and HRI, contribute to translation suppression by detecting virus-induced changes in cellular homeostasis such as nutrient deprivation and accumulation of reactive oxygen species (ROS) or unfolded proteins in the ER. In the cytosol, untranslated mRNAs condense together with numerous RBPs and form SGs (1). Upon infection with certain viruses, innate immune sensors, PKR, regulators of stress and immune sensors, and interferon (IFN)-induced effectors localize in stress granules (SGs) together with viral RNA or DNA, forming a signaling platform (2) that coordinates and potentiates the antiviral response (3,4,5). PKR is at the crossroads of stress and innate immune signaling pathways: PKR interacts with RIG-I, promotes its activation, and amplifies the downstream signaling cascade (6). A similar interaction exists with cGAS (7). PKR promotes MDA5 filament formation, is activated by MDA5 to enhance downstream MAVS signaling (8), and, in turn, can also be activated by MAVS (9). PKR affects pro-inflammatory responses by interacting with TRAFs to activate the NF-κB signaling and potentially the JNK/p38 MAPK pathway leading to AP-1 activation (10), or directly regulates NF-κB activity via phosphorylation of IκB and IKK (11). Finally, PKR is involved in controlling the stability and translation of IFN mRNAs (12). Black arrows indicate signaling pathways; dashed red arrows indicate crossroads between stress response pathways, innate immune signaling, and SGs.

IFN and cytokine production is not only regulated by the abovementioned immune sensing pathways. Several lines of evidence indicate that eIF2α-kinases modulate immune signaling pathways at several levels (Figure 1).

A first mechanism is a direct consequence of translational repression by the eIF2α-kinases, which lowers the levels of key regulatory proteins, especially labile proteins such as IκB, A20, and SHIP-1, leading to elevated activity of NF-κB and IRF3 [189,190,191,192]. A second mechanism involves CHOP, a central uORF-regulated transcription factor induced during the ISR. CHOP causes transcriptional inhibition of peroxisome proliferator-activated receptor γ, which, in turn, is a negative regulator of NF-κB transcriptional activity [193]. Thereby, CHOP indirectly augments NF-κB activity under stress conditions. Furthermore, PKR-mediated translation inhibition was found to be required for full activation of the stress-activated JNK upon poly(I:C) transfection [194].

Independently of its function in translational control, PKR orchestrates a variety of immune and survival pathways, thereby influencing cell fate decisions. For instance, PKR has been implicated in NF-κB activation by directly phosphorylating the NF-κB inhibitor IκB [195]. However, later reports indicated that PKR activates NF-κB signaling indirectly, either in a translation-dependent manner as described above, or by providing a signaling platform via its dsRBD, which recruits various signaling molecules and allows PKR to function as a scaffold independently of its kinase activity. Accordingly, PKR was shown to interact with the β subunit of the IKK complex [196,197,198] as well as with TNF receptor-associated factor (TRAF) 2, TRAF5, and TRAF6 [199]. Additionally, PKR was found to directly interact with components of the RIG-I/MDA5 signaling pathway. For instance, during the very early response to HCV infection, PKR interacts with MAVS and TRAF3, thereby inducing ISG15. Subsequent ISGylation of RIG-I interferes with RIG-I activation and thus, limits IFN induction [200]. In contrast, DHX36-mediated activation of PKR in response to IAV and NDV infection promotes RIG-I activation [201]. PKR was also reported to associate with MDA5 and to stimulate IFN-β production via the MAVS-IRF3/7 signaling cascade in response to VACV infection [202]. In addition, the interaction of PKR with MAVS is suggested to promote PKR activation in response to dsRNA [202,203]. cGAS together with G3BP1 was shown to form a complex with PKR, which is important for cGAS activation and IFN production upon dsDNA exposure. Vice versa, PKR was also activated within the complex [204]. Interactions of PKR with immune-related transcription factors, including STAT1 [205] and STAT3 [206], have also been reported. Finally, PKR coordinates IKK, JNK, and p38 MAPK activity in response to pro-inflammatory stimuli such as TNF-α and IL-1 [207,208].

Another mode by which PKR affects immune signaling is through binding to cis-acting regulatory elements in cytokine mRNAs. Binding of PKR to an element in the TNF-α 3′ UTR was shown to promote TNF-α pre-mRNA splicing [209] and binding to the IFN-γ 5′ UTR appears to suppress IFN-γ translation beyond the effect of PKR on global protein synthesis [210]. PKR has also been implicated in sustaining IFN-α/β mRNA integrity in response to a subset of RNA viruses that activate MDA5 specifically, but the exact mechanism still needs to be clarified [211].

Unlike those identified for PKR, links between the other stress kinases, namely GCN2, PERK, and HRI, and innate immune signaling pathways, are less well understood and are often not studied in the context of virus infections. In vitro, HRI mediates NF-κB activation by phosphorylating IκB [212]. Further to this, HRI was shown to be important in fish for the NF-κB-mediated immune response to RGNNV [160]. Reports about GCN2 focus on its ability to negatively regulate inflammatory responses or positively influence antigen-presentation [213,214,215]. Direct interactions with immune signaling components remain to be investigated. PERK acts as an activator of the JAK1/STAT3 signaling pathway in the context of neuroinflammation [216] and was shown to be important for poly(I:C)-induced TLR inflammatory signaling [217]. On the other hand, PERK activation was reported to induce the degradation of the type I IFN receptor IFNAR1, and thereby inhibits JAK/STAT-mediated production of many ISGs. Viruses such as HCV or vesicular stomatitis virus (VSV) take advantage of that and actively trigger IFNAR1 degradation via PERK activation [218,219]. Further investigations about a possible direct implication of HRI, GCN2, and PERK in innate immune signaling are needed.

Downstream of the eIF2α-kinases, signal transducers also link the ISR to the IFN signaling. ATF4, which is translationally activated upon eIF2α phosphorylation, directly interacts with IRF7, and affects IFN-α/β induction in response to viral infection. This interaction is bidirectional, since IRF7 upregulates ATF4 expression and activity, while ATF4 inhibits IRF7 activation [220]. Moreover, GADD34 expression was proposed to be induced by the MAVS-IRF3/7 pathway in response to poly (I:C) or infection with VSV [192]. Additionally, GADD34 is involved in negative feedback upon TNF-α activation: it recruits CUE domain-containing 2 (CUEDC2) to PP1 and thereby leads to the dephosphorylation of IKKα and β and a decrease in NF-κB activity [221].

Altogether, the translational inhibition of unstable immune-regulatory proteins, as well as interactions of stress kinases or downstream signal transducers with components of the immune signaling cascade, promote the transcription of IFNs and pro-inflammatory cytokines. Hence, induction of the stress response, especially the activation of PKR, upon viral infections strongly contributes to establishing a pro-inflammatory and antiviral state. At the same time, PKR, PERK, ATF4, and GADD34, however, also initiate distinct negative feedback loops in order to fine-tune and possibly locally and temporally restrict IFN production to prevent an overactivation of the innate immune system.

An unsolved question is how antiviral proteins are synthesized when global translation is repressed. While uORFs facilitate translation of mRNAs under such conditions, as exemplified by ATF4 [222], most mRNAs encoding antiviral proteins do not appear to harbor uORFs. GADD34 is induced as a negative feedback regulator upon translational inhibition, leading to dephosphorylation of eIF2α and resumption of translation. Mathematic modeling in combination with flow cytometry experiments suggest that this negative feedback loop contributes to the stochastic expression of IFN in individual cells upon poly(I:C) treatment and VSV infection, whereby transcriptional induction of IFNs during translation-off states is followed by synthesis of IFNs during translation-on states [192,223]. Recently, two different laboratories made another important contribution to the understanding of how antiviral factors are preferentially produced in response to viral infections. They discovered that ribonuclease L (RNase L), which is activated in response to dsRNA, strongly depletes cellular mRNA pools through wide-spread mRNA degradation, but specifically leaves mRNAs encoding IFNs, cytokines, and other defense proteins intact [224,225,226]. The mechanism by which these mRNAs are excluded from RNase L-mediated cleavage remains unclear, but probably involves regulatory sequences, RNA secondary structures, and RBPs that protect individual mRNAs from degradation. Hence, it appears that the combination of increased transcription and selective mRNA stabilization and translation permits the synthesis of antiviral factors under conditions of repressed global translation.

An early link between the ISR, in particular the formation of SGs, and the temporal control of cytokine production comes from studies on the adaptive immune system. In naïve T helper cells, IL-4 mRNA was found to accumulate in SG-like foci in the cytoplasm during T cell priming, concomitant with elevated phosphorylation of eIF2α. The release of these mRNAs then allowed for the rapid production of cytokines during T cell restimulation [227], suggesting that SGs can exert storage and regulatory functions outside of classical stress conditions.

## 5. SGs as Immune Signaling Platforms in Antiviral Defense

While SGs assemble in response to translational shut-off, they are not necessary for translation suppression under stress conditions. Rather, SGs were proposed to function as (i) hubs for modulating local protein and mRNA concentrations; (ii) timers for the stress response, marking a “window of opportunity” during which the stress can be resolved or an apoptotic program will be initiated; (iii) joint assemblies of unfolded proteins and translation complexes that coordinate the activities of the protein synthesis and folding machineries; (iv) storage sites for pre-assembled initiation complexes, allowing for rapid resumption of protein synthesis when cells recover from stress; (v) signaling platforms that connect stress sensors with effectors of immune responses, especially in the context of viral infection [15,16,18,228,229].

**Figure 2:**
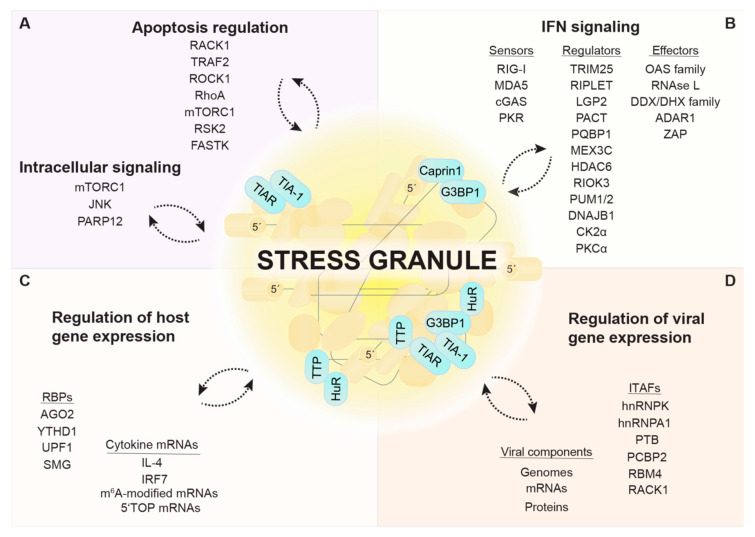
SGs as immune and stress signaling platforms. SGs function as immune and signaling platforms by phase-separating and concentrating regulatory proteins involved in apoptosis induction and other intracellular signaling pathways (**A**) and IFN signaling. (**B**). Certain RBPs and cellular mRNAs (e.g., cytokine mRNAs) preferentially localize in SGs (**C**). Viral components and ITAFs that control viral gene expression are also detected in SGs (**D**). SG composition is highly stress- and cell type-specific. Notably, many components have been detected in SGs under metabolic or environmental stress conditions, while an investigation in the context of viral infection is still missing. The localization and function of some SG components is dependent on the interaction with specific SG core proteins, indicated in turquoise.

Viruses deploy many different strategies to inhibit the formation of SGs, suggesting that SGs serve an antiviral function. In particular, flaviviruses have evolved numerous mechanisms to interfere with SG assembly. The core protein of JEV, for instance, interacts with Caprin1 to relocalize G3BP1 and USP10 to the perinuclear region, thereby preventing SG formation [230]. Other SG components seem to be sequestered by viral RNAs, as reported for TIA1 and TIAR, which bind to the genomic RNA 3′ end of DENV, WNV, and tick-borne encephalitis virus [231,232]. At later stages of infection, flavivirus genomes are degraded by the cellular exoribonuclease XRN1 from the 5′ end up to a compact pseudoknot structure in the 3′ UTR, leading to the cytosolic accumulation of the remaining subgenomic flaviviral RNA (sfRNA) [233,234]. G3BP1, G3BP2, Caprin1, and USP10 are sequestered by sfRNAs, which leads to inhibition of ISG mRNA translation and thus, attenuation of the antiviral response [235]. A similar sequestration strategy was observed for SeV infection, during which trailer RNAs, i.e., small abortive products of viral genome replication, bind TIAR, and inhibit SG formation [236,237,238]. Our own work showed that flaviviruses uncouple the stress response from translation control by blocking both eIF2α phosphorylation and SG assembly, while protein synthesis is still suppressed [58]. In line with this observation, other laboratories reported that the expression of single viral proteins such as Zika virus (ZIKV) capsid, NS3, NS2B-3, or NS4A is sufficient to inhibit SG assembly [239,240,241]. The precise mechanism behind this inhibition remains to be uncovered. In the following, we will focus on the role of SGs within the complex signaling network that controls cellular reprogramming towards an antiviral state (Table 1 and Figure 2).

### 5.1. G3BP1 at the Interface between SGs and the IFN Response

G3BP1 turns out to be a target of particular importance for viruses, which aim to interfere with its function as a SG nucleator and regulator of immune responses. Moreover, G3BP1 appears to facilitate replication of many viruses. Several viral proteins were shown to directly recruit G3BP1 to sites of viral replication, e.g., HCV polymerase NS5B [271,296,297,298], Chikungunya virus nsP3 [299,300], Semliki Forest virus and SINV Nsp3 [301,302], and Junín virus N protein [303]. Murine norovirus inhibits the formation of canonical SGs not only by redistributing G3BP1 together with the NS3 protein to sites of viral replication, but also by modifying the interactome of G3BP1 [304]. Notably, G3BP1 is directly involved in translation of the norovirus genome by associating with the VPg viral cap complex to help ribosome recruitment [305].

In recent years, G3BP1 was also found to promote both the inflammatory and the IFN responses. Overexpression of G3BP1 was shown to induce the formation of SGs to which innate immune factors such as PKR, oligoadenylate synthetase (OAS) 2, and RNase L are recruited, which, in turn, promote the production of certain cytokines such as IL-17, MIP-3a/b, and MCP-5 via activation of the NF-κB and JNK pathways [256]. Independently of its role in SG assembly, G3BP1 was also shown to interact with the RNA sensor RIG-I and positively regulates its downstream signaling [306,307], as well as with the DNA sensor cGAS, thereby enhancing IFN-β production [308]. This might be the reason why several members of the Picornaviridae and related viruses have evolved G3BP1-cleaving proteases, as reported for the Leader protein of foot-and-mouth disease virus, Theiler’s murine encephalomyelitis virus (TMEV) and mengovirus [280,309,310], the proteinase 3C of poliovirus, coxsackievirus B3 and encephalomyocarditis virus (EMCV) [245,311,312,313], as well as the 3C-like proteinase NS6 of feline calicivirus [314]. Hence, G3BP1 and SGs seem to be at the nexus of the stress response and innate immune signaling. The role of SGs as a signaling platform of the antiviral response will be described in the paragraph below.

### 5.2. SGs, a Platform to Initiate IFN Signaling?

In cells infected with IAV lacking the NS1 protein (IAVΔNS1) and thus, unable to counteract PKR, viral RNA was found to co-localize in SGs together with the immune sensors RIG-I and MDA5 as well as other antiviral effectors such as OAS, RNase L, and PKR. A similar redistribution of RIG-I was also observed upon infection with EMCV, adenovirus, and SINV. In these infection models, the inhibition of SG assembly by depletion of essential SG components resulted in a strong attenuation of IFN production and an increase in viral replication. Based on these results, Onomoto et al. proposed the term “antiviral SGs” (avSGs) to indicate that SGs may serve as a platform for the activation of innate immune sensors by viral RNA and the subsequent initiation of the IFN response [242]. While most RNA viruses are sensed by RIG-I alone or by RIG-I and MDA5 together [315], MDA5 is essential for IFN-α/β induction in response to Picornaviridae infection [316,317]. In agreement with the idea of avSGs, MDA5 and dsRNA were found to locate in EMCV-induced SGs at early times post infection. At later stages of EMCV infection, cleavage of G3BP1 by the EMCV protease 3C resulted in SG dissolution concomitant with a reduced IFN-β and cytokine response, which could be rescued expressing a cleavage-resistant G3BP1 mutant [245]. The characterization of avSGs formed upon infection with SeV, again revealed the presence of many antiviral components and SG formation was shown to be important for IFN-β production and viral restriction [263]. Furthermore, late during NDV infection, uncapped viral RNA(+) derived from read-through transcription was found to accumulate together with RIG-I in SGs and trigger RIG-I activation. Consistent with the idea of avSGs, IFN-β induction could be reduced by the disruption of SGs in this model [243].

In contrast to these findings, some studies suggest that SG formation may not be necessary for IFN production upon virus infection. During infection with a mutant mengovirus lacking the Leader protein, for instance, localization of MDA5 to SGs was not a prerequisite for IFN induction. However, it may be important to note that viral dsRNA could not be detected within SGs in this infection model, which may indicate that SGs adopt an antiviral function as platforms for RIG-I or MDA5 activation only when they accumulate viral RNA [95]. Another study showed that disruption of SGs in IAVΔNS1- and SeV-infected cells did not reduce but actually increased RIG-I-mediated IFN-β production [202], which is in direct contradiction with previous observations [242,263]. It is therefore still unclear whether the changes observed in the IFN response ensue from the disruption of SGs as a coordinating platform or are due to the immunomodulatory function of single SG components. Additional experiments based on genetic ablation of other SG components, interference with SG assembly by cycloheximide or ISRIB treatments, or the use of eIF2α S51A mutant cells might be necessary to answer this question

Finally, MDA5 and RIG-I were also detected in SGs formed under other stress conditions such as heat shock and arsenite treatment [95,242], which raises the possibility that MDA5/RIG-I can also assemble with endogenous (e.g., damaged) RNAs in SGs, or that these sensors passively phase separate in SGs.

Whether SGs contribute to the antiviral response upon infection with DNA viruses is even less clear. Upon DNA virus infection, viral DNA is sensed by specialized DNA sensors, among those cGAS. In addition, viral transcripts, cytosolic noncoding RNAs transcribed by the RNA polymerase III, as well as Alu-derived host RNAs can activate MDA5 or RIG-I [318]. In the case of VACVΔE3L, MDA5-mediated IFN-β production was unchanged upon SG dissolution, and rather dependent on the immunomodulatory function of PKR [202]. HSV-1 infection induces formation of SGs, in which dsRNA (whose origin is unclear) was detected. In this infection model, IFN production was induced by the DNA sensing cGAS-STING pathway rather than by MDA5/RIG-I, and preceded PKR activation and SG formation, indicating that SGs are dispensable for cGAS-mediated IFN production upon HSV-1 infection [121]. Interestingly, cGAS was reported to associate with dsDNA, G3BP1, and PKR in a complex and one study claimed the presence in cytoplasmic foci upon dsDNA transfection [204,308]. While it is not clear if these foci represent canonical SGs, they were proposed to be essential for cGAS activation and downstream IFN-β production [204]. Of note, polyglutamine binding protein 1 (PQBP1), identified as a co-sensor of cGAS in the context of HIV infection [319], was shown to localize to SGs [70,255]. Hence, SGs might support cGAS-mediated IFN production under specific conditions or for particular virus types. However, this needs to be further investigated.

Given these discrepancies, the link between SGs and innate immune signaling appears to be complex. Besides the co-localization of innate immune sensors with viral RNAs or DNAs within SGs, the type of virus and the presence or absence of regulatory factors are likely to affect the potential of SGs to serve as platforms initiating the IFN response. It should further be noted that studies so far have mainly focused on the impact of SGs on the production of IFN-β or cytokines such as IL-6, RANTES, and CXCL10. Hence, it would be interesting to investigate how SGs might influence the expression of other IFN types and subtypes, and how this could affect virus replication.

### 5.3. Regulators of the Innate Immune Sensors

RLR activity is tightly controlled by multiple posttranslational modifications and protein interactions. Tripartite motif protein 25 (TRIM25) and Riplet are two examples of E3 ubiquitin ligases, which, according to a current model, mediate consecutive K63-linked ubiquitination of RIG-I. The first ubiquitination event mediated by Riplet leads to the exposure of the RIG-I CARD domains and their subsequent ubiquitination by TRIM25. Thereby RIG-I gets activated, oligomerizes, and initiates downstream MAVS signaling [320]. Both E3 ubiquitin ligases were found to be recruited together with RIG-I to SGs when cells were exposed to poly(I:C) [249]. It is not clear, however, whether co-localization of these proteins within SGs is important for the sequential ubiquitination of RIG-I and downstream signaling events. Using a bimolecular fluorescence complementation assay and super-resolution microscopy in SeV-infected cells, RIG-I was identified in two distinct complexes—RIG-I/TRIM25 and RIG-I/MAVS—with different cellular localization [248]. While TRIM25/RIG-I complexes localized in SGs, RIG-I/MAVS complexes remained attached to mitochondrial membranes. An interaction between MAVS and TRIM25 was barely observed [248], contrasting previous studies in which TRIM25 was proposed to act as an ubiquitin ligase of MAVS [321,322]. RIG-I/TRIM25 complexes further appeared to be in close proximity with mitochondria upon virus stimulation [248], consistent with previous reports about physical contacts between avSGs and MAVS [242,243]. These findings suggest that RIG-I first needs to interact with TRIM25 within SGs to become ubiquitinated and activated before it is released from the complex to activate MAVS at mitochondria. It should be noted that TRIM25 also has a second function: when bound to FAT10, it stabilizes the protein, which acts as a negative regulator of RIG-I and prevents the formation of avSGs under conditions of inflammation [323]. Recent in vivo studies suggest that Riplet alone is sufficient for RIG-I activation [324,325], and confirmed a role of Riplet in modulating the kinetics of RIG-I recruitment into SGs. However, the ability of Riplet to activate RIG-I signaling was not dependent on SG formation [324]. Apart from Riplet and TRIM25, other E3 ubiquitin ligases can mediate RIG-I ubiquitination including MEX3C, an RNA-binding E3 ligase that was found to localize in NDV-induced avSGs and plays an essential role in RIG-I-mediated IFN signaling [244]. Moreover, RIG-I activity is regulated by the activity of several kinases, phosphatases, and acetyl transferases, some of which localize to SGs such as histone deacetylase 6 (HDAC6) [252], PKC-α [251], and the α-subunits of CKII [250].

MDA5 activity is similarly regulated by posttranslational modifications [326]. RIO kinase 3 (RIOK3) is involved in maintaining MDA5 inactive under normal conditions by phosphorylating its C-terminal domain, thereby interfering with MDA5 filament formation on dsRNA and downstream IFN signaling. Upon poly(I:C) exposure, RIOK3 was shown to be partially recruited to SGs, together with MDA5 [246]. DNAJ heat shock protein family (Hsp40) member B1 (DNAJB1), a member of the Hsp40 family, was identified as an interactor of MDA5 that negatively regulates MDA5-MAVS signaling. Upon poly(I:C) treatment, DNAJB1 in conjunction with Hsp70 was found to co-localize with SG markers and the mitochondrial membrane, negatively affecting MDA5 oligomerization and MAVS aggregation [247]. If and how the co-localization of MDA5 together with the DNAJB1-Hsp70 complex or RIOK3 within SGs affects MDA5-MAVS signaling still needs to be investigated.

Laboratory of genetics and physiology 2 (LGP2, DHX58) belongs to the RLR family and acts as a modulatory co-receptor of RIG-I and MDA5 [315]. LGP2 has been shown to localize in SGs upon IAVΔNS1 infection [242]. Moreover, the positive activator of PKR (PACT, PRKRA) was identified as an LGP2 interactor essential for RLR regulation [327], and also shown to localize in SGs upon treatment with hippuristanol [257], a steroid compound that interferes with cap-dependent translation [270]. Interestingly, PACT was also found in a complex with RIG-I and MDA5, positively regulating their downstream signaling [328,329]. In addition, two members of the pumilio RBP family, PUM1 and PUM2, localized to SGs and were found to associate with LGP2 upon NDV infection, enhancing its dsRNA binding ability through conformational changes and thereby increasing the IFN response [253]. Whether co-localization of LGP2 with PACT, PUM1, and PUM2 in SGs is necessary for the regulatory effects on RIG-I and MDA5 activity is currently not clear.

### 5.4. Stress Kinase PKR and Its Regulators

Localization of the dsRNA-sensor PKR in SGs is a prime example of their functional relevance [121,256,330]. PKR is recruited to SGs through its direct interaction with the NTF2 and PXXP domains of G3BP1 [330]. By complex formation with G3BP1 and Caprin1, PKR can be activated in a dsRNA-independent manner. The high local concentrations of all three components in SGs are likely what drive formation of the tripartite G3BP1/Caprin-1/PKR complex. Activated PKR is subsequently released from SGs into the cytosol, where it phosphorylates eIF2α and thereby promotes both translation repression and SG persistence [330]. Hence, SGs are part of a feed-forward loop that amplifies PKR activity and restricts viral replication. This amplification mechanism might explain why so many viruses simultaneously target PKR and SG assembly. One may speculate that the ubiquitin-specific peptidase USP10, which binds to G3BP1 and inhibits SG formation [331], could affect PKR activation and other antiviral signaling events in SGs.

**Figure 3:**
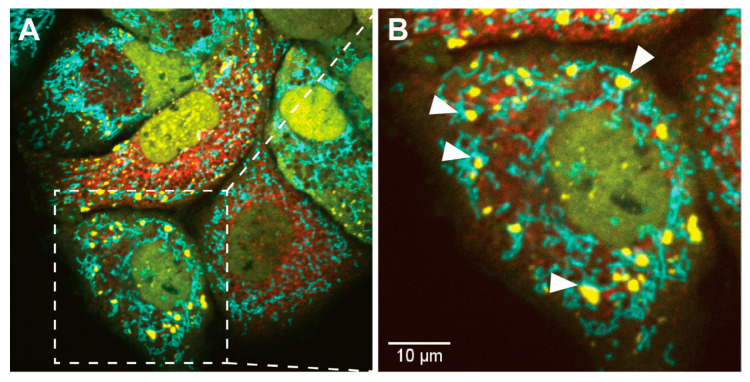
SG localization in close proximity to mitochondria. Shown are live-cell microscopy images of HCV-induced dynamic assembly and disassembly of SGs. (**A**) human hepatocarcinoma Huh7 cells, stably expressing YFP-TIA1 (in yellow) and mTurquoise2-mito [339], a mitochondrial targeting peptide (in cyan), were infected with an HCV-mCherry reporter virus (in red). (**B**) Cropped section. White arrows indicate examples of SGs that are localized in close proximity to mitochondria (scale bar 10 µM).

In contrast, activation of PKR by MAVS was reported to occur outside of SGs prior to its recruitment into SGs [203]. This finding corroborates the idea that PKR is activated in sequential steps and that SGs help to maintain PKR activity at later stages of the infection. Interestingly, PKR was also reported to localize both inside and at the outer membrane of mitochondria [99]. Since SGs and mitochondria are often found in close proximity [248], it is tempting to speculate that PKR shuttles between the two compartments. Accordingly, recruitment of PKR to SGs could affect not only the activation status of PKR within protein complexes inside SGs, but also within protein complexes that specifically form inside or at mitochondria. This scenario is well in line with the tight collaboration between SGs and mitochondria in the RIG-I/MAVS activation pathway described above (Figure 3).

Besides G3BP1 and Caprin1, PACT, transactivation response element RNA-binding protein (TRBP), and P58^IPK^ modulate PKR activity by directly targeting its kinase domain. It is generally thought that PACT stimulates PKR whereas TRBP and P58^IPK^ inhibit PKR activity, although exceptions have been reported and may be cell-context specific [332,333,334,335,336]. The interaction between PKR, PACT, and TRBP is especially important for cellular physiology during viral infections and has implications in stress recovery and the RNA-induced silencing pathway [335,337,338]. However, it is currently unclear whether the recruitment of PKR into SGs affects its interaction with PACT, TRBP, and P58^IPK^. Upon hippuristanol treatment, PACT localizes in SGs where it was proposed to form a complex with argonaute 2 (AGO2) [257]. Furthermore, the SG proteins TIA1 and TIAR were found to suppress PKR activity, although they seem to exert this effect by controlling PACT pre-mRNA splicing, independently of their role in SG assembly [336]. In addition to PACT [257], several modulators of PKR activity, including NFAR1/2 [258,259], adenosine deaminase RNA-specific 1 (ADAR1) [77,262] and Staufen [260], were found to localize in SGs or modulate SGs formation. Further studies are needed to assess whether these PKR regulators relocalize into, or are specifically excluded from, SGs upon virus infection. The differential activation of PKR within different complexes and cellular compartments is likely to dictate the spectrum of PKR targets and thereby decides whether PKR has pro-apoptotic, anti-apoptotic, inflammatory, or translational effects.

### 5.5. Oligoadenylate Synthase and RNase L

Proteins of the OAS family are IFN-induced effectors that are activated by dsRNA binding [340]. OAS proteins polymerize ATPs into 2′-5′-linked oligoadenylates (2-5A), whose lengths vary from dimers to 30-mers [341]. Trimeric or longer 2-5A molecules serve as unique secondary messengers to activate RNase L, which induces RNA degradation [342]. Studies indicate the presence of several proteins of the OAS family within SGs, including OAS1, OAS2, and OAS-like protein (OASL), as well as its mouse homolog OASL1 [159,242,256,267]. OASL, in contrast to the other members of the OAS family, lacks 2-5A synthetase activity but instead, contains ubiquitin-like (UBL) domains [343,344]. OASL was found to co-localize with RIG-I in SGs upon SeV infection and enhances RIG-I-signaling via its UBL domains, mimicking K63-linked ubiquitination. A similar function was suggested for one of the mouse homologues, OASL2 [266]. Interestingly, in the case of DNA virus infections, OASL/OASL2 had the opposite effect, negatively regulating cGAS signaling [345]. OASL1, the second mouse OASL homologue, was similarly shown to be recruited to IAV-induced SGs and to interact with many SG components, including PKR and MDA5 [267]. OASL1 was proposed to act both as a positive and negative regulator of immune signaling. By binding to viral RNA, OASL1 supports MDA5-mediated IFN production early in infection, while its binding to IRF7 mRNA prevents IRF7 synthesis and thereby dampens transcriptional activation of IFN at later time points. However, while SGs are supposed to be important for the activation of the MDA5-MAVS signaling, translational control of IRF7 was not affected by the absence of SGs [267]. Whether the localization of the active 2-5A synthetases OAS1 and OAS2 within SGs has an impact on their function still needs to be clarified.

RNase L activation upon binding of 2-5A results in the cleavage of viral and cellular ssRNAs into small fragments with 5′-hydroxyl and 3′-monophosphate ends [346]. These fragments, which tend to form duplex structures, can activate RIG-I/MDA5-MAVS signaling pathways and enhance the response to virus infection [347,348]. Recently, RNase L was reported to localize in SGs upon transfection of 2-5A. The resulting RNase L cleavage products were found to activate PKR, hence triggering SG formation and subsequent induction of IRF3-mediated IFN response [263]. Contradictory to these findings, earlier reports indicate that RNase L activity reduces PKR protein levels and eIF2α phosphorylation via destabilizing PKR mRNA [349]. In addition, RNase L was shown to inhibit SG assembly or reduce the size of SGs, likely through the global decay of cellular mRNAs, while antiviral mRNAs (e.g., IFN-β mRNA) escape degradation [224,225,226]. Hence, depending on the cell type and the stage of infection, RNase L can apparently enhance or restrict SG formation and the SG-associated antiviral response. In turn, SGs may potentially serve as platforms for interactions between RNase L and viral RNAs, thus enhancing the antiviral activity of RNase L.

### 5.6. Editing of dsRNA by Adenosine Deaminase

ADAR1 is an IFN-induced RNA editing enzyme that catalyzes the C6 deamination of adenosine (A) to inosine (I) within dsRNAs [350]. In cellular RNAs, A-to-I editing weakens RNA duplex structures [351], thereby avoiding the activation of PKR, RIG-I, and MDA5 by self-dsRNAs, most importantly Alu-derived dsRNAs [352]. ADAR1 is recruited to SGs in response to different stresses, IFN treatment, poly(I:C) transfection, and MV and HCV infection [77,245,262,353,354]. Considerable evidence supports the notion that ADAR1 acts as an immune suppressor by promoting the survival and replication of RNA viruses. Wild type MV infection causes induction of IFN-β and subsequent activation of ADAR1. Infection with C-deficient MV, which is not able to counteract PKR, induces SGs in ADAR1 knockout but not in ADAR1-sufficient cells, indicating that ADAR1 antagonizes SG formation [353,354,355]. Thus, ADAR1 sustains viral replication through inhibition of both SG formation and IFN production. However, this proviral effect of ADAR1 activity appears to be virus-specific, since ADAR1 was also found to work as an antiviral immune modulator against HCV infection [356]. Therefore, ADAR1 seems to be a regulator for both SG assembly and antiviral immune responses, yet it remains to be determined whether the recruitment of ADAR1 to SGs influences the decision of whether ADAR1 acts in a pro- or antiviral manner.

### 5.7. Zinc-Finger Antiviral Protein

Zinc-finger antiviral protein (ZAP or PARP13) is an IFN-induced effector protein, which is expressed in two isoforms (ZAP-S and ZAP-L), both lacking poly(ADP-ribose) polymerase (PARP) activity [357]. ZAP is recruited to SGs and participates together with other family members in maintaining SG integrity [264]. In addition, it has a range of different antiviral functions, depending on the factors it interacts with. Acting as an RNA sensor, ZAP was shown to bind to murine leukemia virus (MLV) transcripts, mediating their degradation through recruitment of the RNA exosome machinery. Interestingly, exosome components such as EXOSC5 were found to localize within RNA granules (SGs and PBs) in a ZAP-dependent manner. Similarly, MLV transcripts were recruited [358]. However, it is not clear if the co-localization of these factors into granules is important for viral restriction. Another study found that ZAP was similarly recruited to SINV-induced SGs together with viral RNA. ZAP mutants revealed that recruitment to SGs correlates with ZAP antiviral activity. However, this study also indicated that ZAP interaction partners are important for antiviral function [265]. TRIM25, as an example, was shown to be an essential co-factor for the ability of ZAP to block SINV translation [359]. Beside the exosome, ZAP was further shown to interact with RNA-induced silencing complex (RISC) components such as AGO2, repressing the miRNA ability against antiviral factors, hence mediating an increase in many ISGs [360]. In addition, ZAP-S was reported to interact with RIG-I to strengthen IFN signaling upon IAV and NDV infections [361]. Contradictory to this study, upon knockdown of ZAP-S and exposure with poly(U/UC) RNA, others reported an increase in IFN-β, IFN-λ2, and IFN-λ3 mRNA levels, indicating that ZAP-S has a function in the degradation of those mRNAs, therefore rather in the resolution of the antiviral response. In addition, they claimed that the different cellular localization of both isoforms (with ZAP-S excluded from sites of viral replication) has an impact on their binding preference for host or viral RNA [362]. It would be interesting to investigate how the co-localization of ZAP-S/ZAP-L target RNAs and interactors within SGs shapes ZAP-dependent antiviral functions.

### 5.8. Other DEAD/H-Box Proteins

DEAD/H-box proteins (DDX, DHX) are a large family of helicases involved in multiple cellular processes, which cover nearly all aspects of RNA metabolism [363,364] and include the RNA sensors RIG-I (DDX58) and MDA5 (RH116). DEAD/H-box proteins can be engaged in cytosolic viral RNA sensing, promote or inhibit IFN and cytokine signaling, function as co-factors for other immune sensors and regulators, and control viral replication by protein/protein and protein/RNA interactions. Whether these proteins promote or inhibit viral infections seems to be virus-specific and depend on the cellular context. The helicases DDX1, DDX3, and DDX21, for instance, are frequently required for efficient cytokine and IFN production, whereas DDX19 has been described as a negative regulator of type I IFN production in the context of viral infections [20]. DHX36 (RHAU) was shown to interact with PKR in an RNA-dependent manner, leading to activation of PKR, subsequent SG assembly, and enhanced RIG-I signaling upon IAVΔNS1 and NDV infection [201]. DHX30 was shown to exert an antiviral role by directly interacting with and stimulating ZAP activity against MLV [365]. Proteome analyses of purified SGs induced by oxidative stress confirmed the presence of multiple DEAD/H-box proteins inside SGs, including DDX1, DDX2 (eIF4A), DDX3, DDX6 (Rck), DDX19 (Dbp5), DDX21, DDX47, and DDX50 as well as DHX30 and DHX36 [70]. Localization in SGs could also be confirmed by immunofluorescence microscopy for DDX1, DDX2, DDX3, DDX6, DDX19, and DHX36 [201,268,269,270,271,272,273,274,275,276,277]. Currently, it is not well understood if the immune regulatory functions of these proteins are affected by their localization in SGs, and localization in SGs during viral infections has so far only been addressed for DDX3, DDX6, and DHX36 [201,271,274,366].

For the RNA helicase DDX3, the connection to viral infections and SG assembly has been studied extensively [367]. Furthermore, HDAC6-mediated deacetylation of DDX3 is needed for the maturation of SGs upon stress, a process by which SGs fuse and grow in size [368]. The ability of DDX3 to induce SG assembly was demonstrated to depend on DDX3′s interaction with eIF4E. A DDX3 mutant with impaired eIF4E binding instead did not only show reduced SG assembly but also reduced cell survival upon stress [272]. In the context of HCV infection, recruitment of DDX3 into SGs has a proviral effect. By recruiting DDX3 into SGs, HCV can prevent DDX3 from exerting its positive effect on the translation of PACT mRNA [366]. Moreover, DDX3 was shown to recruit IKKα into SGs concomitant with activation of IKKα. This activation, however, did not induce pro-inflammatory NF-κB signaling, but enhanced proviral lipogenic gene expression [271]. Future studies will have to show how DDX3 mediates IKKα activation in SGs, if this mechanism operates also upon infection with other viruses, and whether this mode of IKKα activation can also exert antiviral effects via the canonical NF-κB signaling.

Finally, DDX6 was shown to co-localize with RIG-I in SGs upon infection with an NS1-deficient Influenza B virus, and promote IFN-β induction [274]. However, the effect of DDX6 on IFN induction seems to be independent of SGs since it was also observed in cells infected with wild type influenza B virus, which inhibits SG formation [274]. Taken together, experimental data for modulation of DEAD/H-box protein activities via recruitment to SGs, especially in the context of viral infections, are scarce and await further investigation.

## 6. Antiviral SG Functions Beyond

In addition to providing an immune signaling platform, SGs have been connected to other antiviral and pro-survival effects (Figure 2). SGs were suggested to contribute to an antiviral state by (i) sequestration of viral RNAs, (ii) clearing viral components through granulophagy, (iii) sequestering RBPs together with viral and cellular mRNAs to modulate gene expression programs, (iv) acting as signaling hubs to coordinate a general stress response, and (v) controlling the initiation of apoptotic programs.

### 6.1. Sequestration of Viral RNAs in SGs or SG-Like Structures

In many cases, SGs appear to sequester viral mRNAs and/or viral genomes to prevent viral replication. Evidence comes from studies using mutant viruses that are no longer able to hinder SG assembly or counteract PKR, as well as from viruses that per se cannot prevent SG assembly. Examples of mutant viruses, for which viral RNAs were found to relocalize in SGs, are VHS-1-deficient HSV-1 and NS1-deficient IAV [121,242,369]. Furthermore, HIV-1 nef mRNA was observed to localize in SGs in a Sam68 mutant cell background [370].

The formation of “antiviral granules” (AVGs) that form in close proximity to viral replication factories and silence viral mRNAs was observed in cells infected with VACV and rabies virus. AVGs form upon PKR activation by viral dsRNA and contain several canonical SG markers, including G3BP1, TIA1, Caprin1, and USP10. Unlike SGs, however, AVGs do not dissolve upon cycloheximide treatment [371,372,373], suggesting that their components are not in exchange with polysomes. Enrichment of viral mRNAs in AVGs is associated with their silencing and the inhibition of viral protein translation. The inhibition of AVG formation by knockdown or knockout of TIA1 instead resulted in increased viral replication [372,373]. Similar cytosolic granules were also observed during infection with TGEV and were found to accumulate both TGEV genomic and subgenomic RNAs together with TIA1 and the polypyrimidine tract-binding protein (PTB). The occurrence of these granules was supposed to either spatiotemporally control viral gene expression or to restrict viral translation and hence, infection [281].

HPIV3 is an example where canonical SGs are induced early during infection, leading to sequestration and silencing of viral mRNAs. However, SG assembly is suppressed at later stages of infection by the HPIV3 proteins N and P, and viral mRNAs are shielded in inclusion bodies for efficient replication [120]. EMCV infection is another example where SGs are induced transiently and sequester EMCV RNA [245]. While sequestration of viral RNAs in SGs is generally thought to represent an antiviral strategy, it is also possible that in some cases, viral replication might benefit from the concentration of viral RNAs in SGs. Given that many viruses including poliovirus, infectious bronchitis virus, mengovirus, and TMEV efficiently prevent the recruitment of viral RNAs into SGs [280,374,375,376], it is reasonable to postulate that SGs primarily serve an antiviral function.

In conclusion, sequestration of viral RNAs within SGs might be a mechanism that applies only to certain viruses to restrict viral replication and spreading. Whether the recruitment of these viral RNAs is an active sorting process or just a consequence of random inclusion is unknown. Generally, whether the presence of viral RNAs in granules or SGs is an antiviral mechanism or a spatiotemporally controlled way to ensure viral replication is unclear.

### 6.2. Granulophagy

SGs are known to be cleared by autophagy, also termed granulophagy [377,378]. Given that LINE- and SINE-derived retroelement RNAs are specifically sorted into SGs and PBs and subsequently cleared by granulophagy [379], this process could help cells to clear viruses and trapped viral RNAs. First indications for such a model came from reports on the role of autophagy in clearing poly(I:C)-induced SGs [267]. Recent work on coxsackievirus A16 revealed that SGs induced early during infection were later cleared by autophagy as a means to suppress antiviral immune signaling [380]. Some viruses hijack the autophagic degradation pathway or use autophagosome-derived compartments to support their replication [381,382]. Infectious bursal disease virus is an example of a virus that subverts the autolysosome for virus assembly and maturation in an acidic environment [383]. It is tempting to speculate that in these cases, granulophagy may also serve a proviral function. In conclusion, there is emerging evidence that viral sequestration and granulophagy might be an effective host mechanism to suppress viral spreading. Furthermore, as it is often the case, some viruses appear to subvert this mechanism to their own advantage.

### 6.3. Recruitment of RBPs and Associated RNAs

During stress, numerous RBPs are known to relocalize into SGs. In the context of viral infections, the recruitment of RBPs that function as ITAFs, controlling the activity of viral and cellular IRESs, and the sequestration of RBPs regulating the expression of cytokine and stress response mRNAs may have a direct impact on the antiviral response [384]. Among canonical and non-canonical ITAFs (see for reviews [384,385]), G3BP1 [69,386], heterogeneous nuclear ribonucleoprotein (hnRNP) A1 [278,387], poly(rC) binding protein 2 (PCBP2), [279,388,389], receptor for activated C kinase 1 (RACK1) [292,390], PTB [280,391], RNA-binding motif protein 4 (RBM4) [282], Hu Antigen R (HuR) [392,393], and hnRNPK [283,394] localize in SGs. Interestingly, the anti-apoptotic Bcl-xL mRNA was found to be sequestered together with its inhibitory ITAFs in SGs, which prevents its translation during osmotic stress [395]. However, there is no experimental evidence that relocalization of ITAFs in SGs would affect the translation of viral IRES-containing mRNAs.

Since SGs are considered to be separated biochemical compartments within the cytoplasm, they might contribute to mounting a stress-responsive gene expression profile by affecting mRNA translation and stability rates. An interesting observation is that cells lacking the ability to form SGs tend to over-react to stress. In such cells, stress-induced mRNAs are induced to higher levels, while repressed mRNAs are more strongly repressed. This led to the proposition that SGs, by recruiting RBPs alone or together with their target mRNAs, may provide a buffering system for changes in gene expression [396]. In addition, the recruitment of mRNAs together with translation initiation factors into SGs is thought to help maintain these mRNAs in a translationally silent state during stress and allow for rapid reinitiation of their translation upon resolution of stress. Moreover, the recruitment of certain mRNAs into SGs was found to correlate with enhanced mRNA stability [397]. It should be noted, however, that this view was challenged by a recent single molecule analysis, which found that mRNAs originating from SGs are translated and degraded at similar rates to their cytosolic counterparts, at least during a harsh condition such as incubation with arsenite [398].

The RBPs TIA1, TIAR, HuR, and TTP are involved in regulating the stability and translation of many mRNAs encoding pro- and anti-inflammatory cytokines. At the same time, these RBPs belong to a group of well-characterized SG proteins [399]. Of particular importance to viral infections, IFN-ß and IFN-γ mRNAs contain AREs, which mediate control of mRNA stability through binding of HuR and TTP [400,401]. Stress-dependent phosphorylation events are known to control both the activity and SG localization of several of these RBPs [186,402]. TTP, for instance, is phosphorylated via the stress-activated p38 MAPK/MK2 cascade, which prevents TTP from binding to AREs, mediating degradation of ARE-containing mRNAs, and associating with SGs [186,188]. While it is intriguing that protein activity and SG localization are controlled by the same phosphorylation event, it is still not clear whether inclusion in or exclusion from SGs per se is relevant for the effector functions as well as the RNA and protein interactions of these posttranscriptional regulators of cytokine expression.

In addition to the sequence-specific RBPs, microRNA (miR) binding and subsequent assembly of the RISC are known to suppress translation and induce the decay of cytokine mRNAs, either in the cytoplasm or after recruitment into PBs. Localization of AGO2 in SGs was found to counteract RISC activity in the cytosol, thus contributing to enhanced mRNA stability [284]. Relocalization of AGO2 from PBs and SGs to lipid droplets is further required for miR-122-dependent HCV replication [297]. In addition, components of the nonsense-mediated decay (NMD) machinery such as UPF1 and SMG1 often localize in SGs. It will be important to determine whether the relocalization of these factors into SGs affects the efficiency of NMD, not least because viral genomes are generally prone to activate NMD due to their multicistronic gene arrangement [403].

Finally, two different sets of transcripts are preferentially recruited to SGs—m^6^A-modified mRNAs [286] and mRNAs containing a 5′-terminal polypyrimidine tract (5′TOP) motif [398]. m^6^A modification of cellular mRNAs was found to change upon infection by DENV, ZIKV, WNV, and HCV, and influence the expression of hundreds of mRNAs that regulate flavivirus infection [404]. Notably, m^6^A modification controls the production and stability of the IFN-β mRNA [405,406] and the translation of the transcription factor Foxo3, an antagonist of ISG transcription [407]. YTHDF proteins, which specifically recognize m^6^A, were found to interact with m^6^A-containing mRNAs at the shell of SGs [286]. Given the role of m^6^A in regulating the expression of innate immune factors, the enrichment of m^6^A-containing mRNAs in SGs may contribute to the control of viral replication. The preferential recruitment of 5′TOP mRNAs in SGs is also interesting. Since these mRNAs encode all ribosomal proteins and several translation factors, their sequestration could exert a sustained suppressive effect on virus replication by reducing the long-term availability of the translation apparatus [26].

### 6.4. SGs as Signaling Hubs to Coordinate the General Stress Response

Approximately 50% of the proteins in SGs are not known to bind RNA but rather contribute to cell signaling during stress. Thus, not only RBPs, but also various enzymes that catalyze posttranslational modifications localize in SGs. These include for example components of mTORC1 complex, JNK and its scaffold protein WDR62, the ubiquitin peptidase USP10, the protein arginine methyltransferase (PRMT) 1 and PRMT5, the glycosyltransferase OGG1, and the poly-ADP-ribose polymerase PARP12 [70,408]. Accordingly, proteins within SGs were found to carry a plethora of posttranslational modifications including phosphorylation [250,409], GlcNac glycosylation [408], sumoylation [410], neddylation [411], ubiquitination [380], and ADP-ribosylation [264].

The localization of enzymes within SGs can have different consequences. First, enzymes localize in SGs to modulate the assembly, disassembly, or composition of SGs. They are needed for SG biology, but SGs, in turn, are unlikely to influence the activity of these enzymes. Second, enzymes are sequestered in SGs as a way of silencing their activity. This may be particularly relevant for low abundance proteins with low SG shuttling rates, leading to efficient separation of the enzyme from its cytosolic or nuclear targets. Third, enzymes are recruited in their active form into SGs, or become activated inside SGs, to participate in signaling pathways. This mechanism is likely to benefit from the high concentration of enzyme and substrate within SGs.

One interesting example in this context is the recruitment of PARP12 into SGs. While the various cellular functions of PARP12 are not fully understood, PARP12 expression is induced by IFN and has an antiviral role that can potentially be modulated in a SG-dependent manner. The antiviral functions of PARP12 have been linked to its ability to bind viral and cellular RNAs, and a possible role in translation control [412,413]. PARP12 might further be involved in ADP ribosylation of AGO proteins within SGs, which potentially results in reduced microRNA activity [264]. Relocalization of PARP12 from the Golgi into SGs was further shown to result in the disassembly of the Golgi compartment and in a block of the anterograde membrane trafficking [287]—a route that many viruses rely on for particle assembly. In contrast, inflammation-induced association between PARP12 and p62/SQSTM1 outside of SGs was found to increase NF-κB signaling [412]. Moreover, PARP12-mediated ADP-ribosylation of ZIKV proteins NS1 and NS3 in the cytosol leads to their degradation and restricts virus replication [414]. Taken together, these findings point to a potential role of SG localization for PARP12 substrate selection and its multiple effects on virus replication.

### 6.5. Apoptosis Control by SGs

Several reports indicate that SGs fulfill important anti-apoptotic functions. An attractive concept is that cells, upon damage or insult, assemble SGs while they attempt to resolve the stressful conditions. Cytosolic phase separation could herein mark this “window of opportunity”, and only if the damage persists and stress cannot be resolved cells would undergo apoptosis [15]. For several types of stress, SGs were found to efficiently sequester RACK1, thereby inhibiting RACK1-dependent activation of the pro-apoptotic mitogen-activated protein kinase MTK1-p38/JNK signaling axis [292]. Similarly, the localization of the small GTPase RhoA and the Rho-associated coiled-coil containing protein kinase 1 (ROCK1) in SGs hinders the induction of JNK-mediated apoptosis [295]. NF-κB-mediated pro-inflammatory signaling—which can also lead to apoptosis—is partially suppressed by eIF4G-dependent recruitment of TRAF2 into SGs during heat stress and arsenite exposure [293]. Furthermore, Astrin-dependent recruitment of mTORC1 into SGs also prevents apoptosis, which may arise from mTOR hyperactivation, upon exposure to arsenite [289]. An anti-apoptotic role was also revealed for the co-localization of TIA1 and the p90 ribosomal S6 kinase 2 (RSK2) in SGs upon oxidative stress [294]. The Fas-activated serine/threonine kinase (FASTK) is a pro-survival factor that also localizes in SGs and controls protein synthesis of the anti-apoptotic factors cIAP-1 and XIAP. Like RSK2, FASTK interacts with and mediates its anti-apoptotic effects by binding to and regulating TIA1 [415], though it is not yet clear if the SG-resident pool of FASTK is critical to its anti-apoptotic function. Finally, DDX3-dependent assembly of SGs was found to negatively correlate with DDX3-mediated induction of the NLP3 inflammasome and subsequent pyroptosis in LPS-challenged macrophages [416]. Notably, the above examples assign an anti-apoptotic role to SGs, indicating that SGs indeed play an active role in opening a “window of opportunity” for cells in distress, by delaying the onset of apoptosis. Most lytic viruses induce premature death of the infected host cell. To prevent this from turning into a disadvantage by limiting viral spread, some have evolved molecular strategies to counteract or delay apoptosis. One example is SeV, in which encoded trailer RNA binds TIAR to limit apoptosis and SG formation [238]. On the other hand, viruses can also actively trigger apoptosis to release progenies during later stages of lytic infections [417]. Whether SGs also serve as a timer function for the life span of virus-infected cells remains, however, to be determined.

## 7. Conclusions

Knowing whether RBPs and cellular or viral RNAs are actively “recruited”, “sequestered”, or just passively “phase separated” into SGs is much more than a matter of semantics—it is key to a mechanistic understanding of how SGs shape host–virus interactions and coordinate signaling events. Evidence for the presence of a specific RNA or protein in SGs largely relies on microscopy analyses, which have recently been complemented by the development of labeling methods, purification protocols, and the use of high-resolution imaging. However, most SG studies are performed in cells exposed to oxidative stress or other harsh conditions, and many aspects of SG biology still need to be addressed in virus-infected cells.

The co-evolution of virus and host can be described as an arms race that needs to be carefully balanced with the consequences of collateral damage. The dynamic nature of SGs gives testimony to this contest: while some viruses effectively suppress SG assembly, others face an initial wave of SGs that they can only curb during later stages of infection. The appearance of oscillating SGs during chronic virus infections is maybe the most stunning strategy by which cells contain “the enemy within” without suffocating from the antiviral response.

It is tempting to speculate that the condensation state, composition, and function of SGs may change over time as viral infections progress. Further studies building on the recent technical advances will be required to mechanistically dissect the antiviral role of SGs and provide a time-resolved understanding of how stress response pathways and innate immune signaling intersect in the context of viral infections. This may eventually allow us to harness the SGs’ “dance with the devil” for antiviral therapeutic approaches against important human pathogens.

## Figures and Tables

**Table 1:** Localization of proteins with antiviral functions in SGs during stress or viral infection.

Protein	ISG	Molecular Function	Cellular Function Related to Antiviral Defense	Stress Condition/Virus	Reference
**Innate Immune Sensors**					
**RIG-I** **(DDX58)**	ISG	Cytosolic RNA sensor; RNA helicase	Detection of viral nucleic acids (short dsRNA with 5′ppp end primarily) Induction of innate immune signaling via MAVS	Arsenite, infections with IAVΔNS1, EMCV, SINV, Adenovirus, NDV	[242,243,244]
**MDA5** **(IFIH1)**	ISG	Cytosolic RNA sensor; RNA helicase	Detection of viral nucleic acids (long dsRNA) Induction of innate immune signaling via MAVS	Arsenite, infections with IAVΔNS1 and EMCV, heat shock	[95,242,245]
**cGAS**	ISG	Cytosolic DNA sensor, Nucleotidyltransferase	Detection of viral nucleic acids (DNA) Production of cGAMP Induction of innate immune signaling via STING	Arsenite, herring testis DNA or IFN stimulatory DNA treatment *	[204]
**Innate Immune Sensor Regulators**					
**RIOK3**		Kinase	Negative regulator of MDA5 via phosphorylation	poly(I:C) transfection	[246]
**DNAJB1**		Chaperone	Negative regulator of MDA5 and MAVS, acting in a complex with Hsp70	poly(I:C) transfection	[247]
**TRIM25 (RNF147, ZNF147)**	ISG	E3 ubiquitin ligase, RBP	Positive regulator of RIG-I via K63-linked ubiquitination	SeV infection, poly(I:C) transfection	[248,249]
**RIPLET** **(RNF135)**		E3 ubiquitin ligase	Positive regulator of RIG-I via K63-linked ubiquitination	poly(I:C) transfection	[249]
**MEX3C**		E3 ubiquitin ligase, RBP	Positive regulator of RIG-I via K63-linked ubiquitination	NDV infection	[244]
**CKII (alpha subunits)**		Kinase	Negative regulator of RIG-I via phosphorylation	Arsenite	[250]
**PKC-α**		Kinase	Negative regulator of RIG-I via phosphorylation	Arsenite, heat shock	[251]
**HDAC6**		Deacetylase	Positive regulator of RIG-I via deacetylation	Arsenite, UV irradiation, CCCP (mitochondrial stress), heat shock	[252]
**LGP2** **(DHX58)**	ISG	Cytosolic RNA sensor; RNA helicase	Detection of viral nucleic acids (dsRNA) Regulator of MDA5 and RIG-I signaling	Arsenite, IAVΔNS1 infection	[242]
**PUM1 and 2**		RBP	Positively affects RNA binding affinity of LGP2	NDV infection, arsenite (PUM2)	[253,254]
**PQBP1**		Protein scaffolding, DNA binding	Co-sensor for cGAS in the context of HIV infection	Arsenite	[255]
**Stress Kinases and Regulators**					
**PKR** **(EIF2AK2)**	ISG	dsRBP, Kinase	Translational control Regulation of innate immune signaling	G3BP1 overexpression, IAVΔNS1 infection, arsenite	[201,242,256]
**PACT**		dsRBP	Positive and negative regulation of PKR, MDA5, RIG-I, and LGP2 activity	Hippuristanol	[257]
**NFAR1/2** **(NF90/NF110)**		dsRBP	Substrates and modulators of PKR activity	poly(I:C) transfection, arsenite	[258,259]
**Staufen**		dsRBP	Regulation of mRNA translation and stability Inhibition of PKR autophosphorylation	Arsenite, thapsigargin	[260]
**IFN EFFECTORS**					
**ADAR1**	ISG	dsRNA-specific adenosine deaminase	Weakens duplex structure of RNA via A-to-I editing, thereby prevents detection of RNA by immune and stress sensors	Arsenite, poly(I:C) transfection, HCV infection	[77,261,262]
**RNase L**	ISG	Endoribonuclease	Degradation of viral RNA and generation of cleavage products that activate RIG-I/MDA5 and PKR; negative regulator of PKR mRNA levels	Arsenite transfection, 2-5A transfection, IAVΔNS1 infection,	[242,263]
**ZAP** **(ZC3HAV1, PARP13)**	ISG	RBP, protein scaffold	Negative regulation of viral transcript levels and their translation Negative regulation of miRNA silencing of antiviral transcripts Positive regulation of RIG-I signaling Negative regulation of IFN-β, IFN-λ2 and IFN-λ3 mRNA levels	SINV infection, arsenite	[264,265]
**OAS1**	ISG	2′-5′-Oligoadenylate Synthetase, dsRBP	Activation of RNase L through the production of 2-5A	Arsenite, IAVΔNS1 infection, 2-5A transfection	[242,263]
**OAS2**	ISG	2′-5′-Oligoadenylate Synthetase, dsRBP	Activation of RNase L through the production of 2-5A	G3BP1 overexpression	[256]
**OASL**	ISG	dsRBP	Positive regulation of RIG-I signaling	SeV infection	[266]
**OASL1**	ISG	dsRBP	Positive regulation of MDA5 signaling Negative regulation of IRF7 translation	poly(I:C) transfection, IAV infection	[267]
**OTHER DEAD/H-BOX PROTEINS**					
**DDX1**		ATP-dependent RNA helicase	Regulation of gene expression (transcription, RNA processing) Activation of NF-κB and IFN signaling pathways Pro- or antiviral role in viral infections	Arsenite	[268,269]
**DDX2 (eIF4A)**		ATP-dependent RNA helicase,	Translation control	Hippuristanol, pateamine, arsenite	[270]
**DDX3**		ATP-dependent RNA helicase, protein scaffold	Regulation of gene expression (transcription, splicing, mRNA export, translation) Activation of IKK and IFN signaling pathways Pro- or antiviral role in viral infections	HCV infection, poly(I:C) transfection, arsenite, sorbitol	[271,272,273]
**DDX6** **(Rck)**		Putative ATP-Dependent RNA Helicase	Regulation of mRNA repression and degradation Pro- or antiviral role in viral infections Enhancer of RIG-I signaling	NS1-deficient influenza B virus, arsenite, heat shock	[274,275]
**DDX19** **(Dbp5)**		ATP-dependent RNA helicase	Regulation of mRNA export Negative regulation of IFN production	Tubercidin, arsenite	[276]
**DHX36** **(RHAU)**		ATP-dependent RNA helicase, unwinding of G4 structures	Regulation of gene expression and genome integrity Activation of IFN signaling	poly(I:C) transfection, IAVΔNS1 infection, NDV infection, arsenite, hippuristanol, heat shock, CCCP	[201,277]
**mRNA REGULATION**					
**hnRNPA1**		RBP/ITAF	Regulation of gene expression (mRNA transport, splicing) Translation control	Arsenite, osmotic stress, heat shock	[278]
**PCBP2**		RBP/ITAF	Translation control	Arsenite, heat shock, DTT	[279]
**PTB**		RBP/ITAF	Regulation of mRNA splicing Translation control	L-deficient TMEV infection	[280,281]
**RBM4**		RBP/ITAF	Regulation of mRNA splicing Translation control	Arsenite	[282]
**hnRNPK**		RBP/ITAF	Translation control	Arsenite, puromycin, sorbitol	[283]
**AGO2**		RBP	RNA-induced silencing	Arsenite, heat shock, hippuristanol	[257,284]
**UPF1**		RBP	Regulation of Nonsense-mediated decay	Arsenite, heat shock	[285]
**SMG1**		RBP	Regulation of Nonsense-mediated decay	Arsenite, heat shock	[285]
**YTHDF1/2/3**		RBP	m^6^A-readerRegulation of gene expression (mRNA splicing, translation, and stability)	Arsenite	[286]
**STRESS RESPONSE**					
**PARP12**	ISG	poly-ADP-ribose polymerase	Regulation of Golgi apparatus homeostasis Translational control Activation of NF-κB signaling pathway	Arsenite, heat shock	[264,287]
**mTOR**		Kinase	Translational control Autophagy Regulation of apoptosis	Arsenite, osmotic stress, heat shock	[288,289,290]
**JNK**		Kinase	Cytokine and stress signaling Regulation of apoptosis	TIA1 or TTP overexpression	[291]
**APOPTOSIS REGULATION**					
**RACK1**		Protein scaffold/ITAF	Regulation of stress signaling and apoptosis	Arsenite, thapsigargin, hypoxia	[292]
**TRAF2**		E3 ubiquitin ligase, scaffold	Regulation of apoptosis Activation of JNK and NFKB signaling	Arsenite, heat shock, puromycin, FCCP	[293]
**RSK2**		Kinase	Coordination of survival and proliferation	Arsenite	[294]
**RhoA/ROCK1**		Kinase	Regulation of stress signaling and apoptosis	Heat shock	[295]
**FASTK**		Kinase, protein scaffold	Regulation of stress signaling and survival	G3BP1 overexpression, arsenite	[71]

* not clear if these granules are canonical SGs. Abbreviations: interferon-stimulated gene (ISG), RNA binding protein (RBP), IRES trans-acting factor (ITAF), carbonyl cyanide m-chlorophenylhydrazone (CCCP), trifluoromethoxy carbonylcyanide phenylhydrazone (FCCP), dithiothreitol (DTT).
